# Targeting TRIB3 and PML-RARα interaction against APL

**DOI:** 10.18632/oncotarget.19442

**Published:** 2017-07-22

**Authors:** Ke Li, Feng Wang, Zhuo-Wei Hu

**Affiliations:** Immunology and Cancer Pharmacology Group, State Key Laboratory of Bioactive Substance and Function of Natural Medicines, Institute of Materia Medica, Chinese Academy of Medical Sciences & Peking Union Medical College, Beijing, China

**Keywords:** α-helical peptide, AML, protein-protein interactions, protein quality control, sumoylation

Extensive studies have led to the identification of protein-protein interaction (PPI) cores and intersections that are critical for the acquisition and maintenance of properties of malignant transformation [1]. With technological advances in PPI modulator discovery and validation of PPI-targeting agents, for instance, several small-molecule PPI antagonists that target the interactions of MDM2/p53, XIAP/caspase-9 and BCL2/beclin1 are being studied in clinical trials for cancer patients; targeting of PPI interfaces as an anticancer strategy has come to a reality gradually. This development is likely to continue in light of the huge market and application prospects [1]. Therefore, identifying new small-molecule PPI modulators have advanced clinical perspective for cancer therapy.

Acute promyelocytic leukemia (APL) is driven by the oncoprotein PML-RARα that induces the differentiation block and the transcriptional repression, and promotes APL-initiating cell self-renewal. Recent studies have shown that PML-RARα also affects PML sumoylation and PML nuclear bodies assembly, which regulate stem cell self-renewal [2]. Combined retinoic acid (RA) with arsenic trioxide (ATO) dramatically improves the prognosis of APL patients and cure most patients. Mechanistically both agents directly target the PML-RARα-mediated transcriptional repression and protein stability which induce promyelocyte differentiation and eliminate leukemia-initiating cells. At the molecular level, RA and ATO bind to the RARα moiety or PML moiety of PNL-RARα respectively. ATO further induces PML/PML-RARα sumoylation and sumoylated PML/PML-RARα interacts with E3 ubiquitin-ligase RNF4, ubiquitin, proteasomes and other sumoylated proteins, and recruits them onto PML nuclear bodies (PML-NBs), where ATO integrates the ubiquitination and degradation of PML-RARα/PML. Despite that combination of RA and ATO makes APL highly curable, relapsed and refractory APL still occur among all prognostic subgroups of APL patients. Therefore, searching for PPIs modulators promoting PML-RARα degradation, based on these two novel agents, may bring about new therapeutic options for APL therapy.

Indeed, we recently report that TRIB3, a member of pseudokinase family, promotes APL progression through stabilization of the oncoprotein PML-RARα and inhibition of p53-mediated senescence [3]. TRIB3 acts as a member of tribbles homolog family that are fundamental regulators of cell cycle, differentiation, metabolism, proliferation, and cell stress, and is involved in chronic inflammatory and malignant disease through their interactions with intracellular signaling and functional proteins [4]. Our previous study revealed that TRIB3 promoted cancer development and progression by interacting with the signaling molecule SMAD3 or a selective autophagy receptor p62/SQSTM1 [5, 6]. An α-helical peptide, which disturbs the interaction of TRIB3 and p62, displays an effective activation of autophagic flux, and significantly suppresses tumor growth and metastasis. In our current study, we find that the elevated TRIB3 expression promotes APL progression and therapy resistance through interacting with PML-RARα. Furthermore, this interaction inhibits sumoylation of PML by impeding the association of sumoylation E2 (UBC9) or sumoylation E3 (PIAS1) enzyme with substrate, and the dissociation of PIAS1 from UBC9-SUMO complexes. Subsequently, the ubiquitylation and degradation of PML-RARα are suppressed by TRIB3 through removal of proteasomes, E3 ubiquitin ligase RNF4 to PML-NBs. Given TRIB3 and PML-RARα/PML interaction is critically involved in the progression of APL, targeting this interaction is a potential therapeutic option against APL.

**Figure 1:**
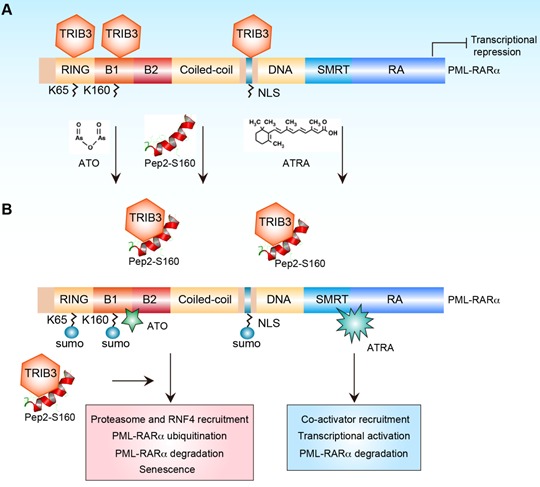
The role of TRIB3 and PML-RARα interaction in PML-RARα-driven APL The elevated intracellular TRIB3 expression can physically interact with the oncoprotein PML-RARα, and this interaction locates in the B-Box1, Ring finger, and the Nuclear Localization Signal (NLS) domain of PML-RARα, and sustains the transcriptional repression of PML-RARα, which promotes PML-RARα-driven APL progression (Panel A). Interruption of the TRIB3 and PML-RARα interaction with a α-helical peptide (Pep2-S160) combined with ATO or ATRA treatment attenuates APL by recruitment of proteasome and RNF4, restoration of PML-RARα sumoylation, ubiquitylation and degradation, and p53 mediated senescence (Panel B). Thus, the TRIB3 and PML-RARα interaction is critically involved in the pathogenesis of PML-RARα-driven APL. Targeting the TRIB3/PML-RARα interaction is a potential therapeutic option for the treatment of APL.

Despite that PPIs act as a novel and highly promising class of drug targets, there are a number of challenges and concerns regarding PPIs targets due to the existence of large interface areas, a lack of deep pockets, and the presence of noncontiguous-binding sites [1]. Thus, a small molecule could hardly dock into the interface surfaces of many PPIs. However, the development of structural studies permits the identification of peptide fragments and amino acid residues which are critical for PPIs. Moreover, mimicking the structure of binding peptides is one of the approaches widely used to design novel PPIs modulators. Interestingly, in this current study we found that TRIB3 mainly interacted with the B-Box1, Ring finger, and the Nuclear Localization Signal (NLS) domain of PML-RARα (Figure 1A). PML-RARα can be sumoylated at 3 lysine residues: K65 in the RING finger domain, K160 in the B1 box domain and K490/497 in the NLS domain. The mutants deleting all 3 consensus motifs for PML-RARα sumoylation show no interaction with TRIB3 at all, which implicated that TRIB3 interacts with the consensus motif for PML-RARα sumoylation. We further screened α-helical peptides with a high affinity for TRIB3 from the PML moiety covering the consensus motif of PML-RARα sumoylation. Among them, S160 peptide shows pharmacological disturbance of the TRIB3 and PML-RARα interaction. When linked with a cell-penetrating peptide identified previously [7], this fused peptide Pep2-S160 attenuates or eradicates APL by restoring the sumoylation of PML/PML-RARα, PML-NBs assembly and p53 mediated senescence combined with ATRA/As2O3 treatment (Figure 1B). Our findings indicate that targeting TRIB3/PML-RARα interaction is a promising alterative or adjunct therapy for APL.

It seems relatively easy to find exquisitely specific and very tight-binding peptides that block PPIs. But the real challenge is to turn these peptides into drugs that are actually effective in patients, for the reasons that peptides have an unstable conformation, do not readily enter cells and are easily destroyed by proteolysis. In hence, at this stage our studies mainly provide a proof-of-concept for targeting the TRIB3/PML-RARα interaction to promote the degradation of the oncoprotein PML-RARα as an attractive therapeutic strategy against APL. With regard to the peptide S160 that disturbing TRIB3/PML-RARα interaction, more efforts including structure modification and optimization should be performed to enhance its cell penetrating ability and stability to make it more druggable.
